# Dark matter in a deep-sea vent and in human mouth

**DOI:** 10.1111/j.1462-2920.2007.01434.x

**Published:** 2007-10

**Authors:** Michael Y Galperin

**Affiliations:** National Center for Biotechnology Information, National Library of Medicine, National Institutes of Health Bethesda, MD 20894, USA.

Summer used to be a slow time for genomics news. This year, even the summer heat failed to stem the influx of new completely sequenced microbial genomes. The latest list (Table 1) includes genomes from a number of environmental bacteria (Chen *et al*., 2007; Nakagawa *et al*., 2007), four methanogenic archaea, as well as the finished genome of the red alga *Cyanidioschyzon merolae*, the first 100% complete eukaryotic genome (Nozaki *et al*., 2007). However, this time the most striking news comes from an unfinished genome, the genome sequence of the first representative of the enigmatic TM7 phylum (Hugenholtz *et al*., 2001). So far, no member of this widespread phylum has been isolated in pure culture and the genome sequence, even an incomplete one, provides the first glimpse into the physiology of this biological ‘dark matter’ (Marcy *et al*., 2007).

**Table 1:** Recently completed microbial genomes (June–August 2007).

Species name	Taxonomy	GenBank accession	Genome size, bp	Proteins (total)	Sequencing centre^a^	Reference
**New organisms**						
*Cyanidioschyzon merolae*	*Eukaryota, Rhodophyta*	AP006483–AP006502	16 546 747 (total)	4775	Univ. Tokyo	Nozaki *et al*. (2007)
*Methanococcus aeolicus*	*Euryarchaeota*	CP000743	1 569 500	1490	JGI	Unpublished
*Methanococcus vannielii*	*Euryarchaeota*	CP000742	1 720 048	1678	JGI	Unpublished
*Candidatus Methanoregula boonei*	*Euryarchaeota*	CP000780	2 542 943	2450	JGI	Unpublished
*Kineococcus radiotolerans*	*Actinobacteria*	CP000750	4 761 183	4497	JGI	Unpublished
		CP000752	12 917			
*Bacteroides vulgatus*	*Bacteroidetes*	CP000139	5 163 189	4065	WashU	Xu *et al*. (2007)
*Flavobacterium psychrophilum*	*Bacteroidetes*	AM398681	2 861 988	2412	INRA – Jouy-en-Josas	Duchaud *et al*. (2007)
*Parabacteroides distasonis*	*Bacteroidetes*	CP000140	4 811 379	3850	WashU	Xu *et al*. (2007)
*Alkaliphilus metalliredigens*	*Firmicutes*	CP000724	4 929 566	4625	JGI	Unpublished
*Bacillus amyloliquefaciens*	*Firmicutes*	CP000560	3 918 589	3693	U. Göttingen	Chen *et al*. (2007)
*Clostridium beijerinckii*	*Firmicutes*	CP000721	6 000 632	5020	JGI	Unpublished
*Clostridium kluyveri*	*Firmicutes*	CP000673	3 964 618	3913	U. Göttingen	Unpublished
		CP000674	59 182			
*Ochrobactrum anthropi*	α-*Proteobacteria*	CP000758–CP000763	5 205 777 (total)	4799	JGI	Unpublished
*Parvibaculum lavamentivorans*	α-*Proteobacteria*	CP000774	3 914 745	3636	JGI	Unpublished
*Sinorhizobium medicae*	α-*Proteobacteria*	CP000738–CP000741	6 817 576	6213	JGI	Unpublished
*Xanthobacter autotrophicus*	α-*Proteobacteria*	CP000781	5 308 934	5035	JGI	Unpublished
			316 164			
*Janthinobacterium* sp. Marseille	β-*Proteobacteria*	CP000269	4 110 251	3697	CNRS-Marseille	Audic *et al*. (2007)
*Actinobacillus succinogenes*	γ-*Proteobacteria*	CP000746	2 319 663	2079	JGI	Unpublished
*Klebsiella pneumoniae*	γ-*Proteobacteria*	CP000647	5 694 894	5187	WashU	Unpublished
*Marinomonas* sp. MWYL1	γ-*Proteobacteria*	CP000749	5 100 344	4439	JGI	Unpublished
*Anaeromyxobacter* sp. Fw109-5	δ-*Proteobacteria*	CP000769	5 277 990	4466	JGI	Unpublished
*Campylobacter curvus*	ε-*Proteobacteria*	CP000767	1 971 264	1931	JCVI	Unpublished
*Campylobacter hominis*	ε-*Proteobacteria*	CP000776	1 711 273	1682	JCVI	Unpublished
*Nitratiruptor* sp. SB155-2	ε-*Proteobacteria*	AP009179	1 877 931	1857	JAMSTEC	Nakagawa *et al*. (2007)
*Sulfurovum* sp. NBC37-1	ε-*Proteobacteria*	AP009179	2 562 277	2466	JAMSTEC	Nakagawa *et al*. (2007)
*Fervidobacterium nodosum*	*Thermotogae*	CP000771	1 948 941	1750	JGI	Unpublished
*Thermosipho melanesiensis*	*Thermotogae*	CP000716	1 915 238	1879	JGI	Unpublished
**New strains**						
*Methanococcus maripaludis* C7	*Euryarchaeota*	CP000745	1 772 694	1788	JGI	Unpublished
*Mycobacterium tuberculosis* F11	*Actinobacteria*	CP000717	4 424 435	3941	Broad Institute	Unpublished
*Bacillus cereus* ssp. *cytotoxis* NVH 391-98	*Firmicutes*	CP000764	4 087 024	3844	JGI	Lapidus *et al*. (2007)
		CP000765	7 135			
*Clostridium botulinum* A str. ATCC 19397	*Firmicutes*	CP000726	3 863 450	3552	Los Alamos	Unpublished
*Clostridium botulinum* A str. Hall	*Firmicutes*	CP000727	3 760 560	3407	Los Alamos	Unpublished
*Clostridium botulinum* F str. Langeland	*Firmicutes*	CP000728	3 995 387	3659	Los Alamos	Unpublished
		CP000729	17 531			
*Staphylococcus aureus* ssp. *aureus* JH1	*Firmicutes*	CP000736	2 906 700	2780	JGI	Unpublished
		CP000737	30 429			
*Staphylococcus aureus* ssp. *aureus* str. Newman	*Firmicutes*	AP009351	2 878 897	2614	Juntendo Univ.	Unpublished
*Coxiella burnetii* Dugway 7E9-12	γ-*Proteobacteria*	CP000733	2 158 758	2125	JCVI	Unpublished
			54 179			
*Haemophilus influenzae* PittEE	γ-*Proteobacteria*	CP000671	1 813 033	1623	Allegheny Institute	Unpublished
*Haemophilus influenzae* PittGG	γ-*Proteobacteria*	CP000672	1 887 192	1670	Allegheny Institute	Unpublished
*Pseudomonas aeruginosa* PA7	γ-*Proteobacteria*	CP000744	6 588 339	6286	JCVI	Unpublished
*Shewanella baltica* OS185	γ-*Proteobacteria*	CP000753	5 229 686	4394	JGI	Unpublished
		CP000754	83 224			
*Yersinia pseudotuberculosis* IP 31758	γ-*Proteobacteria*	CP000720	4 723 306	4324	JCVI	Unpublished
		CP000719	153 140			
		CP000718	58 679			
*Campylobacter jejuni* ssp. *doylei* 269.97	ε-*Proteobacteria*	CP000768	1 845 106	1731	JCVI	Unpublished

a Sequencing centre names are abbreviated as follows: Allegeny Institute, Allegheny-Singer Research Institute, Pittsburgh, PA, USA; CNRS-Marseille, CNRS – UPR2589, Institut de Biologie structurale et Microbiologie, Marseille, France; INRA – Jouy-en-Josas, Unité Virologie et Immunologie Moléculaires, Institut National de la Recherche Agronomique, Jouy-en-Josas, France; JAMSTEC, Japan Agency for Maine-Earth Science and Technology, Natsushima-cho, Yokosuka, Japan; JCVI, J. Craig Venter Institute, Rockville, MD, USA; JGI, US Department of Energy Joint Genome Institute, Walnut Creek, CA, USA; Juntendo Univ., Department of Bacteriology at Juntendo University, Bunkyo-ku, Tokyo, Japan; Los Alamos, Bioscience Division, Los Alamos National Laboratory, Los Alamos, NM, USA; U. Göttingen, Göttingen Genomics Laboratory at the Institute of Microbiology and Genetics, University of Göttingen, Göttingen, Germany; Univ. Tokyo, Department of Biological Sciences, Graduate School of Science, University of Tokyo, Tokyo, Japan; WashU, Washington University School of Medicine, St. Louis, MO, USA.

The TM7 phylum was identified based on the comparison of 16S rRNA genes in samples from a variety of terrestrial (peat bog, activated sludge) and aquatic environments. Fluorescence *in situ* hybridization revealed cells forming long, thick (up to 50 × 4 μm) filaments (Hugenholtz *et al*., 2001). Similar sequences were subsequently detected in deep-sea hydrothermal sediment, humic lake, hypersaline wastewater and even in a marine sponge (Lopez-Garcia *et al*., 2003; Lefebvre *et al*., 2006; Newton *et al*., 2006; Thiel *et al*., 2007). Members of TM7 were also detected in human oral cavity and oesophagus, often associated with necrotizing ulcerative gingivitis, halitosis and periodontitis (Paster *et al*., 2002; Brinig *et al*., 2003; Kazor *et al*., 2003; Pei *et al*., 2004). Still, no representative of the TM7 was ever obtained in a pure culture. Genome sequencing of TM7 was made possible by capturing individual cells in a specially designed microfluidic device, followed by DNA amplification and sequencing (Marcy *et al*., 2007). Although the genome size and the number of encoded proteins could not be reliably estimated, the genome assembly produced a total of 2.86 Mb containing 3245 predicted genes. Some of the predicted genes did not have known homologues, while those that did had relatively low sequence identity to genes from known phyla. These observations further confirmed that the analyzed genes came from a representative of a new phylum. Mapping the predicted genes onto the metabolic pathway map suggested that the TM7 isolate was able to perform glycolysis, the tricarboxylic acid cycle, nucleotide biosynthesis and some amino acid biosyntheses. Obviously, the incomplete genome did not allow identification of the missing pathways that might give some clues to the reasons why members of TM7 refuse to grow in pure culture. Anyway, this work represents a significant step towards characterization of these fascinating organisms.

Another major news was successful transformation of *Mycoplasma capricolum* cells with full-length chromosomal DNA from *Mycoplasma mycoides* by a group at the J. C. Venter Institute (Lartigue *et al*., 2007). The efficiency of transformation (referred to as ‘genome transplantation’ by the authors) reached one recipient per 150 000 cells. This is a significant technical accomplishment that opens new possibilities for gene manipulation in the framework of the so-called ‘synthetic biology’.

As mentioned above, scientists at the University of Tokyo, Japan, revised the previously reported genomic sequence of the hot-spring red alga *C. merolae* (Matsuzaki *et al*., 2004), filled all of the 46 remaining gaps, sequenced the 34 remaining chromosomal ends, and reported the first eukaryotic nuclear-genome sequence that is 100% complete (Nozaki *et al*., 2007). The total genome of *C. merolae* is 16 728 945 nucleotides (nt) in size and consists of 20 linear chromosomes with a total of 16 546 747 nt, circular chloroplast genome of 149 987 nt, and a circular mitochondrial genome of 32 211 nt. Each chromosome encodes between 102 and 484 proteins, for a total of 4775, the chloroplast DNA encodes 208 and the mitochondrial DNA encodes 34 proteins. The small size of the *C. merolae* protein set, coupled with the fact that only a small fraction (∼0.5%) of these 5017 genes contain introns, makes this thermophilic (45°C) alga a perfect model organism for studying all kinds of eukaryotic proteins.

For the past 8 years, *Deinococcus radiodurans* remained the only highly radioresistant bacterium with a completely sequenced genome, which severely limited the use of comparative genomics to analyze the resistance mechanisms. The scientists at the US Department of Energy Joint Genome Institute (JGI) have now completed genome sequencing of another radioresistant bacterium, *Kineococcus radiotolerans*, which opens the possibility for meaningful genome comparisons. *Kineococcus radiotolerans* is an aerobic actinobacterium isolated from a highly radioactive waste at the Savannah River Technology Center in Aiken, South Carolina (Phillips *et al*., 2002). Like *D. radiodurans*, this bacterium is highly resistant both to the ionizing γ-radiation and to desiccation. Cells of *K. radiotolerans* produce an orange carotenoid pigment and move by means of polar flagella. The genome encodes all key components of the chemotaxis machinery, including 22 methyl-accepting chemotaxis sensor proteins, by far the largest number found in any actinobacteria. The high resistance of *K. radiotolerans* to dessication suggests that related bacteria might be widespread in arid desert environments.

Although human intestine is not typically perceived as a subject of environmental studies, scientists at the Washington University have embarked on an extensive project aimed at characterizing the microbial diversity in the human intestinal tract, the Human Gut Microbiome (see http://genome.wustl.edu/hgm/HGM_frontpage.cgi). In the previous column we have discussed completion of the genome of the intestinal methanogenic archaeon *Methanobrevibacter smithii* (Samuel *et al*., 2007). The same group has now released complete genomes of two members of the phylum *Bacteroidetes* that are prominently represented in the distal gut of healthy humans, *Bacteroides distasonis* (recently reclassified as *Parabacteroides distasonis*) and *Bacteroides vulgatus* (Xu *et al*., 2007). This paper (which is freely available online) describes a detailed comparison of *P. distasonis* and *B. vulgatus* genomes with the previously sequenced genomes of two other gut symbionts, *Bacteroides fragilis* and *Bacteroides thetaiotaomicron*, and analyzes the role of lateral gene transfer and gene duplication in the adaptation of *Bacteroides* spp. to the gut environment.

One more representative of the *Bacteroidetes* is the widespread fish pathogen *Flavobacterium psychrophilum*, whose genome description (Duchaud *et al*., 2007) was published a month after the release of the genome sequence of the closely related soil bacterium *Flavobacterium johnsoniae*. *Flavobacterium psychrophilum* infects salmon and trout causing haemorrhagic septicaemia, referred to as ‘rainbow trout fry syndrome’, in young fish and severe necrotic lesions called ‘cold-water disease’ in adult fish. In accordance with its name, *F. psychrophilum* grows best at 15°C and is most deadly at temperatures in the 3–15°C range. This bacterium is capable of forming biofilms that can survive in stream water for several months. Accordingly, the genome sequence revealed a cluster of genes involved in the biosynthesis of exopolysaccharides. It also encodes a protein similar to cyanophicin synthase, suggesting that *F. psychrophilum* is capable of storing amino acids, which could contribute to its long-term survival outside of the fish host. The *F. psychrophilum* genome encodes a number of virulence factors, including various proteases, cytolytic toxins and adhesive proteins. Analysis of these virulence factors and other surface proteins should help identify potential vaccine candidates to protect farmed salmon and rainbow trout against infection by *F. psychrophilum.*

The current list (Table 1) includes 10 genomes of low G+C Gram-positive bacteria (*Firmicutes*), two of which represent the genus *Bacillus*, six come from the family *Clostridiaceae* (including three environmental isolates and three new strains of the food-borne pathogen *Clostridium botulinum*) and the remaining two come from new strains of the opportunistic pathogen *Staphylococcus aureus*.

*Bacillus amyloliquefaciens* is an soil bacterium that often colonizes plant rhizosphere, promoting plant growth and suppressing plant pathogens. The plant growth-promoting effect has been attributed to the extracellular phytase activity (degradation of inositol hexaphosphate), which provides the plant with phosphate (Idriss *et al*., 2002). In addition to phytase, *B. amyloliquefaciens* secretes numerous amylases, glucanases and proteases, as well as antibacterial and antifungal compounds. The sequenced strain FZB42 encodes several polyketide synthases, two of which has been shown to be responsible for the synthesis of the polyketide antiobiotics bacillaene and difficidin (Chen *et al*., 2006).

*Bacillus cereus* ssp. *cytotoxis* is a food-borne pathogen, whose genome revealed a significant degree of divergence from the typical *B. cereus* (Lapidus *et al*., 2007). Based on these comparisons, the authors suggest that it should be reclassified as a new species *Bacillus cytotoxicus*.

*Alkaliphilus metalliredigens*, a member of the family *Clostridiaceae*, has been isolated from leachate ponds at the US Borax company in Boron, California, using an enrichment for the ability to reduce Fe(III) in anaerobic conditions at alkaline pH values (Ye *et al*., 2004). *Alkaliphilus metalliredigens* is a strict anaerobe that could tolerate up to 1.5% sodium tetraborate (Na_2_B_4_O_7_) and grew using Fe(III)-citrate, Fe(III)-EDTA, Co(III)-EDTA or Cr(VI) as electron acceptors; yeast extract or lactate served as electron donors. Growth during iron reduction occurred over the pH range of 7.5–11.0 with optimum at pH 9.5, at temperatures ranging from 4°C to 45°C. These properties make *A. metalliredigens* an attractive candidate for bioremediation of metal-contaminated alkaline environments.

Interestingly, another anaerobic iron-reducing bacterium with a recently sequenced genomes belongs to an entirely different phylogenetic lineage, the δ-*Proteobacteria*. *Anaeromyxobacter* strain Fw109-5 has been isolated from an uranium-contaminated subsurface sediment in Oak Ridge, Tennessee (van Landschoot and de Ley, 1983). Although it is an anaerobe, it tolerates microaerophilic conditions and uses acetate, lactate and pyruvate as electron donors and Fe(III) or nitrate as electron acceptors.

*Clostridium beijerinckii* strain NCIMB 8052 is also a strict anaerobe of potential use in biotechnology. It is a soil isolate that ferments a wide range of carbohydrates (pentoses, hexoses, starch and others) to acetate, butyrate, lactate and other products, including valuable solvents acetone, butanol and isopropanol. Analysis of *C. beijerinckii* genome and its comparison with the genome of the closely related solventogenic bacterium *Clostridium acetobutylicum* is expected to provide insight into the mechanisms of solventogenesis and pave way to designing more efficient producers of acetone and butanol suitable for industrial use.

The γ-proteobacterium *Actinobacillus succinogenes*, isolated from the bovine rumen (Guettler *et al*., 1999), is yet another microorganism with potential use in biotechnology. It metabolizes a wide range of sugars (including glucose, fructose, xylose, lactose, and cellobiose), producing succinate, which is a precursor for a number of useful chemical compounds (Zeikus *et al*., 1999).

The γ-proteobacterium *Klebsiella pneumoniae* is best known as an opportunistic human pathogen that causes pneumonia and urinary tract infections in hospital settings and in immunocompromised patients. However, it is a widespread environmental organism, commonly found in soil and water habitats. Distinctive features of *Klebsiella* cells include an extracellular polysaccharide capsule and the ability to fix nitrogen. The sequenced genome comes from a multiple antibiotic-resistant strain *Klebsiella pneumoniae* ssp. *pneumoniae* MGH 78578 that was isolated in 1994 from a pneumonia patient.

The γ-proteobacterium *Marinomonas* sp. strain MWYL1 was isolated near the North Norfolk, England, from the root surface of the salt marsh grass *Spartina anglica*. This grass, as well as some microalgae, produces the osmoprotective compound dimethylsulfoniopropionate, which *Marinomonas* sp. MWYL1 can use as sole carbon source. Metabolism of dimethylsulfoniopropionate produces dimethylsulfide, which is released into the air (Ansede *et al*., 2001) and represents a major contribution to sulfur cycling in the marine environment. Products of dimethylsulfide oxidation in the atmosphere act as cloud condensation nuclei and are largely responsible for forming the cloud cover over the oceans, affecting the climate wordwide (Simó, 2001). The mechanism of dimethylsulfide formation was recently resolved (Todd *et al*., 2007); genome analysis of *Marinomonas* MWYL1 could clarify the regulation of this process.

The four sequenced members of the ε-subdivision of the *Proteobacteria* nicely represent the diversity of this group. *Campylobacter curvus* and *Campylobacter hominis* are gastric pathogens closely related to the better-known *Campylobacter jejuni*, whose genome was recently re-annotated (Gundogdu *et al*., 2007). In contrast, *Nitratiruptor* sp. strain SB155-2 and *Sulfurovum* sp. strain NBC37-1 have been isolated from the deep-sea vents in the Iheya North hydrothermal field, Japan. These bacteria are chemolithoautotrophs that use hydrogen, sulfide, elemental sulfur or thiosulfate as electron donors and oxygen or nitrate as electron acceptors. They are representative of the microbial ‘dark matter’ in the vicinity of the vents, where ε-proteobacteria comprise a significant fraction of the total microbial population (Nakagawa *et al*., 2005). Surprisingly, genome comparisons showed that vent bacteria share with pathogenic ε-proteobacteria a number of genes that had been previously identified as virulence factors (Nakagawa *et al*., 2007). These include genes responsible for N-linked glycosylation, hydrogenase and several other genes. The authors suggest that *Campylobacter*- and *Helicobacter*-like pathogens evolved from free-living ε-proteobacteria, similar to *Nitratiruptor* sp. and *Sulfurovum* sp.

For the past several years, the early branching bacterial phylum *Thermotogales* was represented by a single complete genome of *Thermotoga maritima* (Nelson *et al*., 1999). With an increased focus on microbial diversity, JGI has recently launched a new project aimed at obtaining genome sequences of seven more representatives of this interesting phylum. The genome of *Thermotoga petrophila*, the first one generated by this project, was released earlier this year. The JGI has now released genomes of two more members of the *Thermotogales*, *Fervidobacterium nodosum* strain Rt17-B1, isolated from a hot spring in New Zealand, and *Thermosipho melanesiensis* strain BI429, which was isolated from the gills of a deep-sea vent hydrothermal mussel, *Bathymodiolus brevior*, from the Lau Basin in the South-western Pacific Ocean (Antoine *et al*., 1997). Comparison of the genomes of hot-spring and marine isolates of *Thermotogales* is expected to shed light on the mechanisms of survival in high-pressure marine environments and allow re-assessing the degree of lateral gene transfer from archaea, which in *T. maritima* was estimated to reach 20% of all genes (Nelson *et al*., 1999).

In other genomics news, Jon Hobman, Charles Penn and Mark Pallen of the University of Birmingham have stirred the pot by publishing a paper with the provocative title ‘Laboratory strains of *Escherichia coli*: model citizens or deceitful delinquents growing old disgracefully?’, which states, *inter alia*, that ‘that microbiology's chief idol has feet of clay’ (Hobman *et al*., 2007). Despite somewhat hyped rhetoric, this paper makes a number of valid points, mentioning that *E. coli* K-12 has undergone numerous passages on rich media and cycles of mutagenesis and is hardly representative of either the ancestral *E. coli* or the current environmental and ‘enteropathogenic, enterotoxigenic, enteroinvasive, enterohaemorrhagic, enteroaggregative and diffusely adherent’ strains. In what might be particularly relevant to the subject of this journal, the authors warn against ‘assuming that any models of global regulation or metabolic flux can be generalized to *E. coli* in a state of nature’, look forward to obtaining hundreds, if not thousands, of genome sequences of naturally occurring relatives of *E. coli* K-12 and welcome ‘the bright new, pluralist, genome-saturated “eco-evo” future of *E. coli*’. Thus, aside from the title (and section subtitles), there seems to be very little in this paper to argue about. However, from the genome analysis point of view, it appears that the authors have overlooked a major drawback in our description(s) of *E. coli* K-12, namely the fact that at least one-third of its genes still have unknown (or poorly characterized) function (Riley *et al*., 2006). For example, considering the very similar sets of signal transduction proteins encoded in *E. coli* K-12 and in all other *E. coli* genomes sequenced to date, one has to conclude that we still have only a vague understanding of the functions of its 30 histidine kinases, 29 diguanylate cyclases and/or c-di-GMP-specific phosphodiesterases and two predicted Ser/Thr protein kinases (M.Y. Galperin, in preparation). *Escherichia coli* K-12 still represents our best hope to achieve a complete understanding of the genome of a free-living bacterium and deserves to be treated as such.

In addition to the deliberately provocative comment on *E. coli*, Mark Pallen got involved in another controversy, this time through no fault of his own. About a year ago, Pallen teamed up with Nick Matzke, an evolutionary biologist at the National Center for Science Education in Oakland, California (http://www.natcenscied.org/), to produce a wide-ranging analysis of the evolution of bacterial flagella and refute the claims of proponents of the ‘intelligent design’ on the ‘irreducible complexity’ of that organelle [Pallen and Matzke, 2006; see also the Panda's Thumb weblog (http://www.pandasthumb.org/) and the paper by Scott and Matzke (2007) on the history of the ‘intelligent design’ movement]. In April 2007, Renyi Liu and Howard Ochman also published a paper (freely available online) aimed at refuting the ‘intelligent design’ views on flagellar origin. Liu and Ochman (2007a) compared flagellar proteins from various bacterial genomes using pairwise blast searches with the blast2seq program (Tatusova and Madden, 1999) and detected a certain degree of sequence similarity between nearly all types of proteins. Although in many cases the similarity levels were not statistically significant (unless the low-complexity filtering was deliberately switched off), Liu and Ochman interpreted their results as an evidence of common origin (= homology) between all flagellar proteins, even those that had been known to have different three-dimensional structures and were obviously non-homologous. Those shaky blast results were presented in a form of a ‘network of relationships among flagellar core proteins’, which conveyed an aura of infallibility that must have swayed gullible reviewers and editors of *PNAS*. This paper attracted a positive comment in ScienceNOW (Cutraro, 2007) but was met with a barrage of criticism, including numerous postings on the Panda's Thumb website and T. taxus blog (http://ttaxus.blogspot.com/2007/05/jcvi-evolutionary-genomics-journal-club.html) suggesting that what is true in the paper by Liu and Ochman (2007a) is not new, and what is new is not true. In addition, a recent paper by Doolittle and Zhaxybayeva (2007) questioned the validity of the phylogenetic analysis in that paper. In response, Liu and Ochman published a correction, admitting switching off the low-complexity filter and using a more permissive 9 × 10^−4^ cut-off *E*-value instead of the 10^−4^ value given in the original publication, but claiming that ‘These errors do not affect the conclusions of the article’ (Liu and Ochman, 2007b). This story is interesting not only because *PNAS* has published a deeply flawed paper, something that has happened previously in other prestigious journals, particularly when dealing with ‘hot’ topics. Rather, this case illustrates the caveats of automated sequence analysis, which can only be trusted if the results pass a ‘sanity check’ by a well-trained biologist. Unfortunately, perfunctory sequence analysis has already caused a number of major blunders (Iyer *et al*., 2001) and is likely to generate many more. [Full disclosure: the author was the editor of the original blast2seq paper (Tatusova and Madden, 1999) and is one of the authors of a paper on the possible origin of flagellar ATPases (Mulkidjanian *et al*., 2007)].

Finally, Minoru Kanehisa and colleagues at the University of Kyoto reported an analysis of 191 completely sequenced genomes, aimed at answering a key question: are there any additional directly encoded unusual amino acids besides selenocysteine and pyrrolysine? As these two amino acids (respectively, the 21st and 22nd ones) are both encoded by stop codons, the genomes were inspected for the conservation patterns in the vicinity of the predicted stop codons (Fujita *et al*., 2007). This search failed to find new conserved contexts, which suggested that the 23rd amino acid either has a very limited phylogenetic distribution or does not exist at all.
